# Occurrence of Ticks in Cattle in the New Pastoral Farming Areas in Rufiji District, Tanzania

**DOI:** 10.1155/2016/3420245

**Published:** 2016-11-21

**Authors:** Kamilius A. Mamiro, Henry B. Magwisha, Elpidius J. Rukambile, Martin R. Ruheta, Expery J. Kimboka, Deusdedit J. Malulu, Imna I. Malele

**Affiliations:** ^1^Central Veterinary Laboratory, TVLA, P.O. Box 9254, Dar Es Salaam, Tanzania; ^2^Directorate of Veterinary Services, MLDF, P.O. Box 9152, Dar Es Salaam, Tanzania; ^3^District Veterinary Office, P.O. Box 40, Utete, Rufiji, Tanzania; ^4^Vector & Vector Borne Diseases Institute (VVBD), P.O. Box 1026, Tanga, Tanzania

## Abstract

Ticks and tick-borne diseases plus trypanosomosis are a constraint to cattle rearing in Tanzania. Rufiji district was not known for important ticks infesting cattle because inhabitants were not engaged in keeping livestock. Not only has settlement of pastoralists and cattle in Rufiji increased the number of cattle but also cattle have been the source of bringing in and spreading of ticks. This study investigated tick species that have been introduced and managed to establish themselves in the new livestock farming areas in cattle in Rufiji. Tick distribution study was undertaken in three villages of Chumbi ward seasonally in 2009, 2011, and 2012. The identified ticks were* Amblyomma variegatum* (56.10%)*, Rhipicephalus evertsi* (10.25%),* R. microplus* (27.40%), and* R. appendiculatus* (6.19%) out of 12940 ticks. Results indicate that ticks are present in the new livestock settlement areas. The occurrence of ticks is correlated with the recent settlement of cattle in the district.

## 1. Introduction

Ticks and tick-borne diseases and trypanosomosis and tsetse fly are major constraint to cattle rearing in Tanzania. Intensive tick surveys conducted between 1955 and 1961 provide the basic information on distribution of Tanzanian tick species. According to Yeoman and Walker [1], there are 8 tick genera with 60 identified species. However only 4 genera with 9 species,* Boophilus decoloratus*,* Rhipicephalus appendiculatus*,* Amblyomma variegatum*,* Rhipicephalus evertsi*,* Rhipicephalus microplus*,* Amblyomma lepidum*,* Rhipicephalus pravus*,* Amblyomma gemma,* and* Hyalomma albiparmatum,* characterize cattle tick population of Tanzania. The first 5 of these tick species are principle vectors of some of the most economically important tick-borne diseases of cattle in Tanzania.

Large numbers of indigenous cattle are mostly owned by pastoralists and agropastoralists and account for 98% of cattle population in the country. The grazing land for these animals in areas that is home to many pastoralists is no longer sufficient due to the increase in number of cattle and human population. As a result some of the pastoralists from these areas are currently migrating with their animals opting to settle in other areas of Tanzania where there is an ample grazing land for their animals. Such areas are in the eastern and southern part of Tanzania.

For many years, most areas in eastern Tanzania such as Rufiji district in Coast region have been keeping very few numbers of cattle. This is due to the fact that people living in these areas are not engaged in keeping livestock. Tick surveys conducted by Yeoman and Walker [1], provided information of tick distribution in few areas where very few numbers of cattle were being kept.

Settlement of pastoralists and their animals in some areas of Rufiji district in recent years has not only increased the number of cattle population but also the cattle have been the source for bringing in and spreading ticks of economic importance into the areas. The species of ticks that managed to establish themselves in these new areas together with tsetse fly species present transmit diseases to the animals. In this paper we present results of tick that has managed to establish themselves in the new livestock farming areas of Rufiji in cattle present in the areas.

## 2. Materials and Methods

### 2.1. Description of the Area

Rufiji is one of the six districts of Coast region covering an area of about 14,500 sq km. It lies between latitude 7.47–8.03°S and longitude 38.62–39.17°E with overall altitude of less than 500 metres above sea level. The climate is tropical with cool and dry season from June to September and hot and dry season from December to March. Long rainy season lasts from March to May and short rainy season from October to December. Short rains are unpredictable with variation from year to year. The overall mean temperature is 28°C.

Vegetation is characterized by tropical forests and grasslands. The district has Rufiji River which is a prominent feature that divides the district into two halves. Furthermore Kilwa and Rufiji districts border Selous Game Reserve to the western part. Land utilized for grazing is estimated to be 90,000 ha out of 482,430 ha suitable for grazing.

### 2.2. Sample and Data Collections

#### 2.2.1. Tick Collections

The district veterinary officer (DVO) assisted in providing information about areas where pastoralists from different areas of the country have settled with their animals. Longitudinal study was conducted seasonally in Chumbi ward in 2009, 2011, and 2012 for investigating ticks species. The ward was selected because of high density of cattle introduced from different areas of Tanzania compared to other wards in the district. Chumbi ward comprises three villages namely Chumbi, Kiwanga, and Muhoro.

Ticks were collected from 11 herds. One- to two-month visit intervals were made during the period of rainy season (March–May) and dry season (June–November). During each visit adult ticks were collected from 3-4 head of cattle randomly selected from the herd kraal after the consent was given by each owner in all villages. Collected ticks were preserved in 70% alcohol; however the collected immature stages, nymphs and larvae, were excluded from counting and identification of ticks. The adult ticks were identified in the laboratory according to published keys [2, 3] using a stereo microscope. As these animals were managed by the owners themselves, they were sometimes sprayed with acaricides for tick control.

#### 2.2.2. Data Analysis

Collected data on identified tick species, season and village were entered into Microsoft excel software where they were coded before being analysed by statistical analysis software (SAS). A two-way ANOVA was conducted to detect the effect of season and village on the tick species. Ticks count was used as dependent variable while village, season, and tick species were used as independent variables. Means were separated at 95% confidence interval and the difference was considered significant at 5% in all statistical tests.

## 3. Results

**Figure 1:**
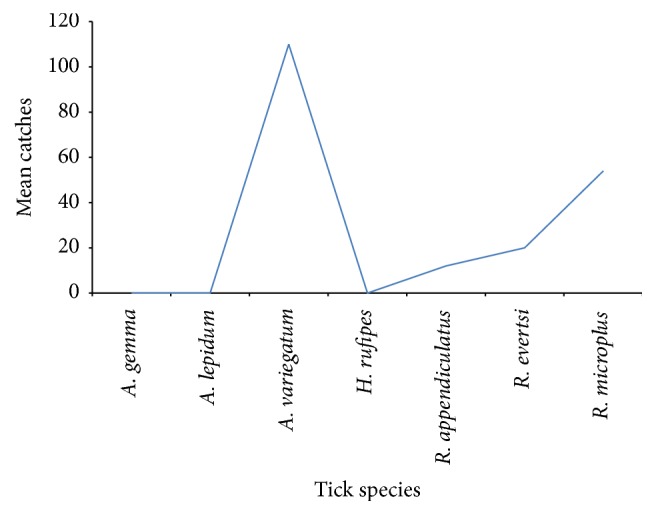
Mean ticks species for the villages of Rufiji district.

*Tick Species Collected and Identified*. A total of 12,940 ticks were collected seasonally from cattle, between 2009, 2011, and 2012 which accounts to the overall mean tick collection of 27.99 ± 3.14. Mean catches for each species are shown in Figure 1. There was high significant difference between tick species (*P* < 0.0001).

The mean catch for* A. variegatum *was the highest (56.6%) followed by* R. microplus* (27.3%),* R. evertsi* (10.2%), and* R. appendiculatus* (6.1%). The less common species were* A. gemma* (0.02%),* Hyalomma rufipes* (0.02%), and* A. lepidum* (0.02%). Neither season (*P* = 0.5981) nor its interaction with species (*P* = 0.7898) and village (*P* = 0.8650) had significant effect on the mean tick count.

**Figure 2:**
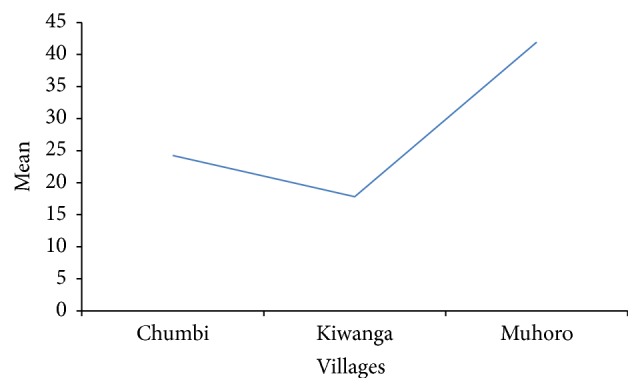
Mean ticks count from villages of Chumbi ward.

**Figure 3:**
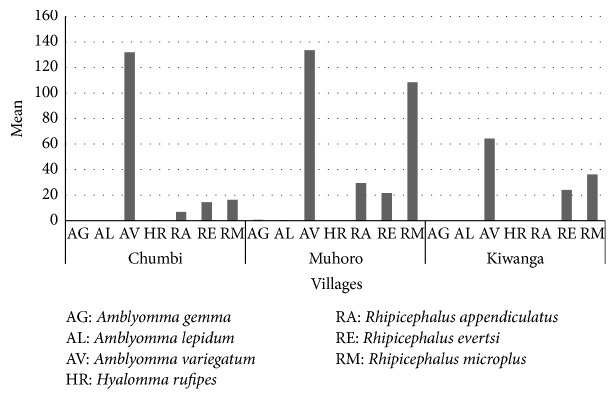
Mean tick species caught seasonally in the three villages of Chumbi ward.

**Figure 4:**
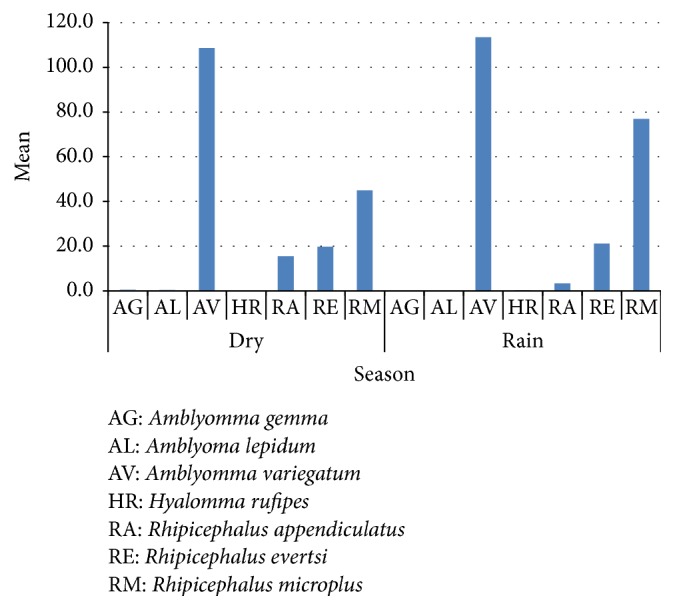
Mean tick species caught seasonally.

Villages had significant effect on the ticks species collected (*P* = 0.0168); ticks collected in Muhoro village were 50% of the total ticks (Figure 2). Further analysis of the interaction between species and villages showed high significant effect on the ticks count (*P* = 0.0042).* R. microplus* of Muhoro and Kiwanga villages was significantly different from the species collected from Chumbi village.* A. variegatum* collected in Kiwanga were significantly different from the ticks of other villages.  Figure 3 shows tick species found in each village. All tick species except* Hyalomma rufipes* were present in Muhoro village; again all tick species except* A. lepidum* were present in Chumbi village.* A. variegatum, R. evertsi, *and* R. microplus *were found in all three surveyed villages.* A. variegatum* was collected in all seasons in high numbers followed by* R. microplus* though the mean count of this species was high during the rainy season (Figure 4).

## 4. Discussion

### 4.1. Tick Species Collected and Identified

Seven tick species were encountered in the new cattle settlement areas of Rufiji, namely,* Amblyomma variegatum*,* Rhipicephalus (B) microplus*,* Rhipicephalus evertsi*,* Hyalomma rufipes*,* Amblyomma gemma*,* Amblyomma lepidum,* and* Rhipicephalus appendiculatus* (Figure 1). These species are widely spread in other areas of Tanzania. More ticks were collected at Muhoro followed by Chumbi compared to Kiwanga. Their distribution and abundance in an area can be related to factors such as climate, vegetation, host density, and grazing habits [4].

The occurrence of these tick species in some areas of Rufiji district to which no livestock were kept before suggests that the species were introduced in these areas by the infested cattle and other livestock brought in by the pastoralists. It appears now that some of tick species introduced have managed to establish themselves, for instance,* Amblyomma variegatum,* which was collected from cattle in the widest range of habitats and assumed to be the most catholic tick species in Tanzania [5]; earlier documented reports indicate that the occurrences of the species in Lindi and areas of Rufiji were negligible with prevalence of 0–0.25. However in this study, the prevalence of* A. variegatum* was 56.10% for seasonal collection during the study period. In early 2007, the country saw a rapid increase in the number of livestock settled in Rufiji district, of the Coastal Region of Tanzania, following the evacuation of livestock from Usangu and Ihefu areas which were declared conservation areas and key water sources for hydroelectric power generation [6]. Many pastoralists opted to settle in the Coastal Region, which has a low human population density, hence ensuring ample grazing land for their animals. Settlement of pastoralists and their animals in the district has also been associated with pressures of land use in the northern circuit of the country [7] as a result; the animal populations in Rufiji district has increased rapidly as a result of this eviction from the wetland sources from 20,000 head of cattle in 2005/2006 to about 140,000 by mid 2008/2009. The updated tick occurrences reported by [5] were conducted between 1998 and 2001 as part of a robust integrated TTBD control regimen, in all 21 regions of the Tanzanian mainland and on Mafia Island in order to update the data on both tick distribution and TBD prevalence in preparation for a National TTBD control strategy based on present-day ecological and epidemiological knowledge of ticks.* A. variegatum *was collected in both seasons during this study (Figure 4).

*A. gemma* was reported to be restricted to central Tanzania, an arid/semiarid bushland/wooded and bushed grassland areas with shorter drought periods and bimodal rainfall [5]; however, during this study, it was found in Rufiji though in low numbers accounting to 0.02% of the total number of ticks collected.

*R. microplus and R. evertsi* were present on cattle during the collection schedules done during the dry season between June and November and rainy season between March and May (Figure 3). All of these species are important vectors of tick-borne disease pathogens reported in Tanzania [8] and elsewhere [9]. Although the study reported by [5] indicated that changing livestock policies, unrestricted livestock movement, and a continuous change in climatic/environmental conditions in Tanzania have brought about only limited changes in the distribution patterns of* R. appendiculatus, R. pravus,* and the three* Amblyomma* species investigated; it remains to be seen that the assumed limited changes in the long run coupled with environmental and/or climate changes will have an impact on the distribution of ticks as demonstrated in this study. Although posterior probability of occurrences of* R. appendiculatus* was 0–0.2 [5], in this study, the prevalence of 6.20% was recorded. The result indicates that* R. appendiculatus* can maintain itself under certain conditions and probably occur seasonally. The spread of* R. appendiculatus* (and ECF) from Lake Victoria basin southwards has also been linked before with migrant agropastoralists from the north-south movement from Sukuma land (Lake Victoria basin) to the Usangu plains (Southern highlands) and all the way to Mtwara [10, 11].

*R. microplus* is a normal species present in coastal areas of Tanzania [1, 4]. It seems that the coastal environment is favourable for this species to maintain itself. The coastal environment is also likely to be favourable for* A. variegatum* and* R. evertsi* which were encountered together with* R. microplus* (Figure 3) and are becoming common species infesting cattle in the pastoralist settlement areas. Earlier studies [12] reported that, except for extremely cold and dry areas,* Rhipicephalus (B) microplus* has extended its distribution range and is now present in all the northern regions of Tanzania and that high suitability is currently recorded for most of the previously nonoccupied areas. The temperature in Rufiji is around 28°C; hence it would support the existence of the species adapted to such temperatures and lower altitudes.

*Rhipicephalus microplus* occurs in areas with an estimated mean rainfall of 58 mm and there was no record of the species in Rufiji [12]; however in our study 27.4% were collected out of 12940 ticks. The very few numbers of* H. rufipes*,* A. gemma,* and* A. lepidum* collected (Figure 1) indicate that climatic conditions and probably other unknown factors in Rufiji district are not favourable for these species which were found infesting cattle introduced from other areas of Tanzania to be able to establish and adapt to the new climatic conditions.

### 4.2. Importance of Collected Ticks in Rufiji

*R. appendiculatus* collected in Rufiji transmits* Theileria parva* which is a causative agent of East Coast fever (ECF), whereas* Anaplasma marginale* causes anaplasmosis and is associated with* R. evertsi* which was one of the ticks identified in the areas. Each of these two tick species are principal vectors of these diseases.

From the present surveys, ticks of cattle appear to be present in the new established cattle settlement areas of Rufiji. It is important for the livestock farmers to be aware and use effective chemicals and drugs to control the vectors. This study indicates that movement of animals in traditional pastoralist system is one of the factors for vector spread and establishment. Hence better land use and planning should be encouraged in order to mitigate factors behind movement of livestock in search of pastures as a result of overgrazing in former areas coupled with poor range land management which cannot support increasing number of cattle in the former pastoralist tradition areas.
